# Comparative cytogenetic study of three *Macrolophus* species (Heteroptera, Miridae)

**DOI:** 10.3897/CompCytogen.v9i4.5530

**Published:** 2015-09-29

**Authors:** Ana Maria Jauset, Eva Edo-Tena, Cristina Castañé, Nuria Agustí, Oscar Alomar, Snejana Grozeva

**Affiliations:** 1 University of Lleida, Department of Crop and Forest Sciences. Alcalde Rovira Roure, 177, 25198 Lleida (Spain); 2 IRTA, Ctra. Cabrils Km 2, 08348 Cabrils (Barcelona), Spain; 3 Institute of Biodiversity and Ecosystem Research, Bulgarian Academy of Sciences, 1 Tsar Osvoboditel Blvd., 1000 Sofia, Bulgaria

**Keywords:** *Macrolophus*, Miridae, Heteroptera, karyotype, sex chromosomes, achiasmate meiosis, sex chromosome pre-reduction, sperm morphology

## Abstract

*Macrolophus pygmaeus* (Rambur, 1839) (Insecta, Heteroptera, Miridae) is a predator of key vegetable crop pests applied as a biocontrol agent in the Mediterranean region. *Macrolophus pygmaeus* and *Macrolophus melanotoma* (A. Costa, 1853) are cryptic species with great morphological similarity which results in their misidentification and negative consequences for the conservation of their populations on greenhouse and outdoor crops. In order to find out specific markers for their separation we studied the karyotype, male meiosis and heterochromatin composition of these species and additionally of a third species (as a reference one), *Macrolophus costalis* Fieber, 1858. We demonstrate here that all the three species share achiasmate male meiosis and sex chromosome pre-reduction. On the other hand, the species differ in karyotype, with 2n=28 (26+XY) in *Macrolophus pygmaeus*, 2n=27 (24+X_1_X_2_Y) in *Macrolophus costalis*, and 2n=34 (32+XY) in *Macrolophus melanotoma*, and heterochromatin distribution and composition. In addition, the species differ in sperm morphology: sperm cells of *Macrolophus costalis* are significantly longer with longer head and tail than those of *Macrolophus melanotoma* and *Macrolophus pygmaeus*, whereas sperm cells of *Macrolophus melanotoma* have a longer tail than those of *Macrolophus pygmaeus*. All these characters can be used as markers to identify the species, in particular the cryptic species *Macrolophus melanotoma* and *Macrolophus pygmaeus*.

## Introduction

The Miridae are the largest family of true bugs (Heteroptera, Cimicomorpha) with approximately 10000 species described (Schuh 1995). Cytogenetical data are presently available for about 200 species (Ueshima 1979, Nokkala and Nokkala 1986, Grozeva 2003, Grozeva et al. 2006, 2007, Grozeva and Simov 2008a, b, 2009, Kuznetsova et al. 2011). The mirid bugs share some cytogenetic characteristics with all the Heteroptera: they possess holokinetic (or holocentric) chromosomes and most of them are characterized by an inverted sequence of reductional and equational division of the sex chromosomes (post-reduction) in male meiosis (Ueshima 1979). On the other hand, they have some unique chromosomal characteristics. Chiasmata are absent in male meiosis, the achiasmate meiosis being of a collochore type (Nokkala and Nokkala 1986, Kuznetsova et al. 2011). In the three hitherto studied *Macrolophus* Fieber, 1858 species, *Macrolophus costalis* Fieber, 1858, *Macrolophus pygmaeus* (Rambur, 1839) and *Macrolophus geranii* Josifov, 1961, both autosomes and sex chromosomes divide pre-reductionally during the achiasmate male meiosis (Grozeva et al. 2006, 2007).

The species from the present study, *Macrolophus costalis*, *Macrolophus melanotoma* (A. Costa, 1853), and *Macrolophus pygmaeus*, occur on a variety of plant species in the Mediterranean region. *Macrolophus pygmaeus* is an efficient predator of several key vegetable crop pests in Europe produced commercially and used widely as a biocontrol agent (Alomar et al. 2002, 2006, van Lenteren 2003, Messelink et al. 2014). *Macrolophus pygmaeus* and *Macrolophus melanotoma* are cryptic species with great morphological similarity which results in their misidentification and negative consequences for the conservation of their populations on greenhouse and outdoor crops. In order to find specific markers for their separation we here studied the karyotype, male meiosis and heterochromatin composition of these species and additionally of a third species, *Macrolophus costalis*. The species have recently been separated based on differences of their genetic profiles, cuticular hydrocarbon composition and on the fact that interspecies crosses do not produce viable progeny (Martinez-Cascales et al. 2006, Gemeno et al. 2012, Castañé et al. 2013). *Macrolophus costalis* can be easily distinguished morphologically from *Macrolophus pygmaeus* or *Macrolophus melanotoma* by the black dot on the scutellum, but it was included in our study as a reference species. In earlier cytogenetic studies (Grozeva et al. 2006, 2007), karyotype of two of the three species here examined was reported. Such characters, as highly asymmetric karyotype (2n=24+X_1_X_2_X_3_Y) with two extra-large autosome pairs and interstitial distribution of C-heterochromatin in them (Grozeva et al. 2006), provide excellent cytogenetic markers to distinguish *Macrolophus costalis* from other *Macrolophus* species. The karyotype of *Macrolophus pygmaeus* (2n=26+XY) is asymmetric, as in *Macrolophus costalis*, but with different number of autosomes and a simple XY sex chromosome system (Grozeva et al. 2007). The species share sex chromosome pre-reduction, but can easily be differentiated by their karyotype and pattern of C-heterochromatin distribution.

Sperm morphology is significant in fertilization (Evans and Simmons 2008). Franco et al. (2011) have recently shown that the species in which sperm competition occurs also displayed the longest sperm length.

With the aim of distinguishing between the cryptic *Macrolophus* species, both karyotype and male meiosis were studied for the first time in *Macrolophus melanotoma* and reinvestigated in *Macrolophus pygmaeus* and (as a reference species) in *Macrolophus costalis* using standard chromosome staining and fluorochromes DAPI and CMA_3_. In addition, morphology of sperm cells was examined in each of the three species.

## Material and methods

### Insects

Males and females of *Macrolophus costalis*, *Macrolophus pygmaeus* and *Macrolophus melanotoma* were collected in Catalonia, NE of Spain, in the vicinity of Mataró (Barcelona) (41.556 North, 2.475 East) from *Cistus albidus* Linnaeus, 1753, commercial tomato fields and *Dittrichia viscosa* (Linnaeus) Greuter, respectively. Colonies from collected individuals were set-up under controlled conditions (25 ± 1°C, 70 ± 10% RH and L16:D8 photoperiod) on tobacco plants (*Nicotiana tabacum* Linnaeus, 1753) with *Ephestia kuehniella* Zeller, 1879 (Lepidoptera, Pyralidae) eggs as a prey (Agusti and Gabarra 2009a, b). All *Macrolophus* specimens were preliminarily identified following Josifov (1992). However, due to morphological similarity between *Macrolophus melanotoma* and *Macrolophus pygmaeus*, their identification was additionally tested by conventional PCR using methodology and specific primers described in Castañe et al. (2013).

### Karyotype

The abdomen of 20 *Macrolophus pygmaeus*, 23 *Macrolophus melanotoma* and 13 *Macrolophus costalis* males were placed in 3:1 fixative (96% ethanol-glacial acetic mixture) and the thorax in 70% ethanol for later species identification by DNA analysis (Castañe et al. 2013). Dissected gonads were squashed in a small drop of 45% acetic acid. The cover slips were removed by the dry ice technique. Slides were dehydrated in fresh fixative (3:1) and air dried. Part of the preparations was stained using Schiff-Giemsa method of Grozeva and Nokkala (1996) to check the number of chromosomes and their behaviour in meiosis. For other slides, DNA- binding fluorochromes, GC-specific chromomycin A_3_ (CMA_3_) and AT-specific 4’-6’-diamino-2-phenylindole (DAPI) were applied following Schweizer (1976) and Donlon and Mafenis (1983), with minor modifications as described in Kuznetsova et al. (2001).

Chromosomes were analyzed using light/fluorescent microscopy (Axio Scope A1 – Carl Zeiss Microscope) at 100× magnification and documented with a ProgRes MFcool – Jenoptik AG digital camera. All cytogenetic preparations and remains of the specimens are stored at the Institute of Biodiversity and Ecosystem Research, BAS in Sofia.

### Sperm morphology

In every species, sperm cells from 10 males (other than those used for the karyotype analysis) were measured following Franco et al. (2011). On a slide, one drop of Beadle saline solution (128.3 mM NaCl, 4.7 mM KCl, 23 mM CaCl_2_) was added to the male abdomen. The seminal vesicle was extracted and opened in 20 μl of saline solution to allow the sperm out. The sperm were diluted carefully with the aid of a fine needle, and then one drop was transferred and smeared across a microscope slide, allowed to dry and rinsed. Sperm cells were analyzed using a Leica DM 4000 light microscope. Twelve sperm cells per individual were measured (head and tail length) at 400x under dark field using the QWin 6.0 (Leica Microsystems, Germany) software package. In order to reduce measurement variation, each component was measured five times for each sperm cell. Data were analyzed by a one-way ANOVA and means separation by Tukey multiple range test.

## Results

### Karyotype

#### *Macrolophus costalis*, 2n=27 (24+X_1_X_2_Y)

Published data: 2n=28 (24+X_1_X_2_X_3_Y) (Grozeva et al. 2006)

**Figures 1–6.**
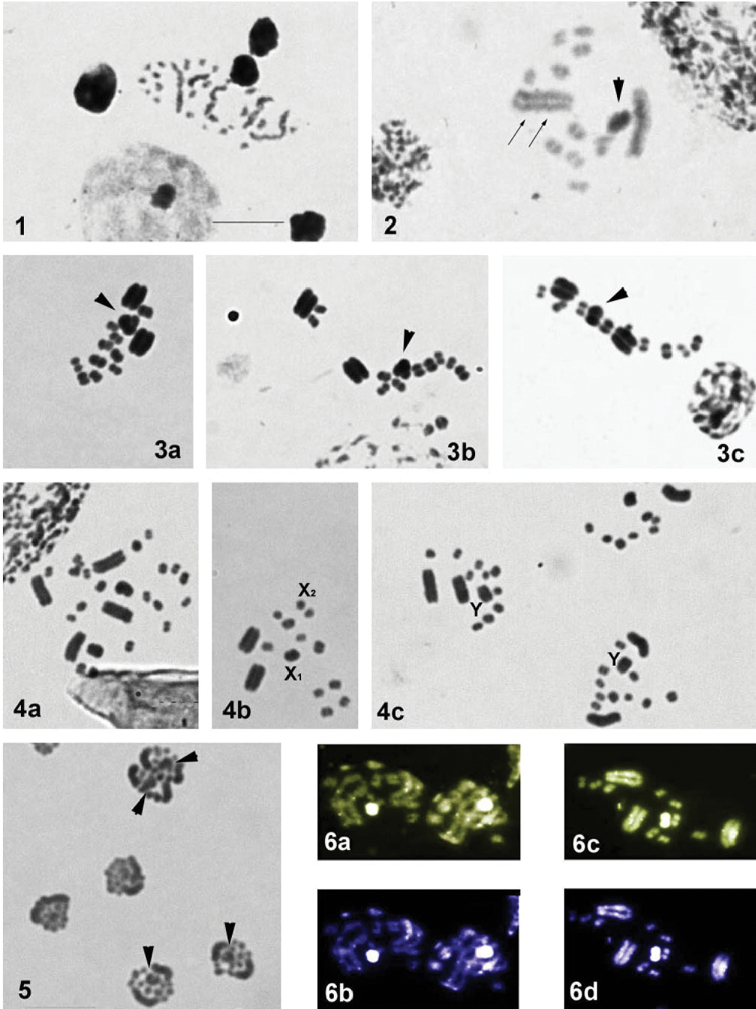
Male meiosis in *Macrolophus costalis*. (**1–5** conventional staining **6** fluorochrome staining: **6a, c** CMA_3_ **6b, d** DAPI) **1** spermatogonial metaphase **2** condensation stage **3a–c** metaphase I **4a–c** metaphase II **5** telophase **6a, b** condensation stage **6c, d** metaphase I. Sex-chromosomes are indicated by arrowheads. Heterochromatin blocks are indicated by arrows. Bar = 10 µm.

Spermatogonial metaphases consisted of 5 large (incl. Y) and 22 similar in size chromosomes (incl. two X) (Fig. 1). In meiosis, condensation stage was most abundant and showed 12 autosomal bivalents plus a positively heteropycnotic sex chromosome body (Fig. 2). Size differences between the bivalents were observed. The complement included two extremely large bivalents, four to five times the size of the other 10 similar size bivalents. Bivalents consisted of parallel-aligned homologous chromosomes without chiasmata, i.e. the male meiosis was achiasmate. After the Schiff-Giemsa staining, it was easy to see that conspicuous interstitial heterochromatic bands in both large bivalents divide them into the three almost equal parts. At metaphase I (MI), the sex chromosomes were seen either as a trivalent (Fig. 3a, b) or as a bivalent (Fig. 3c). They clearly segregated at anaphase I (Fig. 4a) resulting in two types of metaphase II, with 12 autosomes plus two X chromosomes (Fig. 4b) and with 12 autosomes plus the Y (Fig. 4c). Male meiosis was hence pre-reductional both for autosomes and sex chromosomes. After second division, every cell produced daughter cells possessing either 2X or Y chromosome respectively (Fig. 5). Thus, the chromosome formula of male *Macrolophus costalis* was determined as 2n=27 (24+X_1_X_2_Y) in contrast to 2n=28 (24+X_1_X_2_X_3_Y) earlier reported in Grozeva et al. (2006).

After staining with fluorochromes, bright DAPI- and CMA_3_- positive bands were observed in the same locations on the larger autosomal bivalents and sex chromosomes (Fig. 6a–d).

#### *Macrolophus pygmaeus*, 2n=28 (26+XY)

Published data: 2n=28 (26+XY) (Grozeva et al. 2007)

**Figures 7–11.**
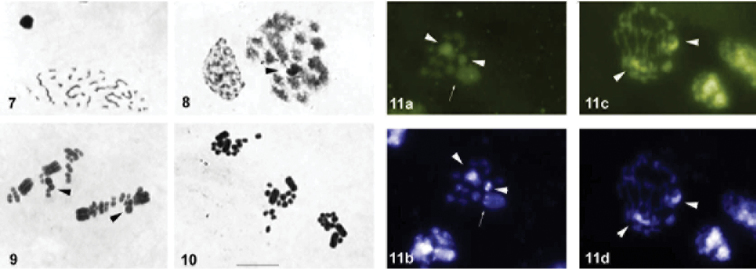
Male meiosis in *Macrolophus pygmaeus*. (**7–10** conventional staining **11** fluorochrome staining: **11a, c** CMA_3_ **11b, d** DAPI) **7** spermatogonial metaphase **8** condensation stage **9** metaphase I **10** metaphase II **11a, b** metaphase I **11c, d** anaphase. Sex-chromosomes are indicated by arrowheads. CMA_3_/DAPI signals are indicated by arrows. Bar = 10 µm.

Spermatogonial metaphases consisted of 5 large (incl. X) and 23 similar size chromosomes (incl. Y) (Fig. 7). In meiosis, condensation stage showed 13 autosomal bivalents and a positively heteropycnotic sex chromosome body (Fig. 8). The complement included two extremely large bivalents (~five times the size of the others), and eleven bivalents similar in size. The bivalents consisted of parallel-aligned homologous chromosomes; chiasmata were absent and the male meiosis was achiasmate. MI was nonradial (i.e., the autosomes did not form a ring), and the sex chromosomes formed a pseudobivalent (Fig. 9). The sex chromosomes segregated at AI resulting in two MII cells each with 14 chromosomes (13 + X or Y) (Fig. 10). Male meiosis was hence pre-reductional both for autosomes and sex chromosomes. Thus, the chromosome formula of *Macrolophus pygmaeus* was confirmed as 2n=28 (26+XY) in line with that earlier reported in Grozeva et al. (2007).

After staining with fluorochromes, bright DAPI- and CMA_3_ -positive bands were observed on both sex chromosomes (Fig. 11a–d). In addition, a weak DAPI- positive / CMA_3_ -negative signal was registered in a telomere of one of the larger bivalents (Fig. 11a, b).

#### *Macrolophus melanotoma* 2n=34 (32+XY)

Published data: absent

**Figures 12–17.**
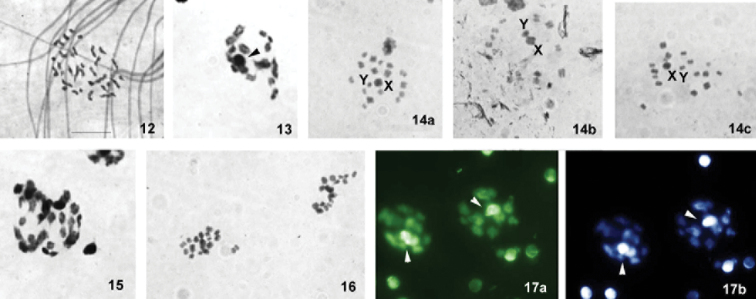
Male meiosis in *Macrolophus melanotoma*. (**12–16** conventional staining **17** fluorochrome staining: **17a** CMA_3_ **17b** DAPI) **12** spermatogonial metaphase **13** condensation stage **14a–c** metaphase I **15** anaphase I **16** metaphase II **17** condensation stage. Sex-chromosomes are indicated by arrowheads. Bar = 10 µm.

At spermatogonial metaphase, there were 34 chromosomes (Fig. 12) gradually decreasing in size and the sex chromosomes were difficult to distinguish. At meiotic condensation stage, autosomal bivalents consisted of parallel lying homologs, and the sex chromosomes appeared as a heteropycnotic body (Fig. 13). At MI, there were 16 autosomal bivalents and X and Y chromosomes (Fig. 14a–c). Note that both sex chromosomes were occasionally placed in the center of a ring formed by autosomal bivalents (Fig. 14b). The autosomal bivalents constituted a decreasing size series and the X was more than twice the size of the Y. As a result of pre-reductional division of sex chromosomes at AI (Fig. 15), two types of MII (Fig. 16) raised, both with 17 chromosomes while with X or Y chromosome respectively.

After staining with fluorochromes, bright DAPI- and CMA_3_ bands were observed on the sex chromosomes (Fig. 17).

### Sperm morphology

**Figure 18.**
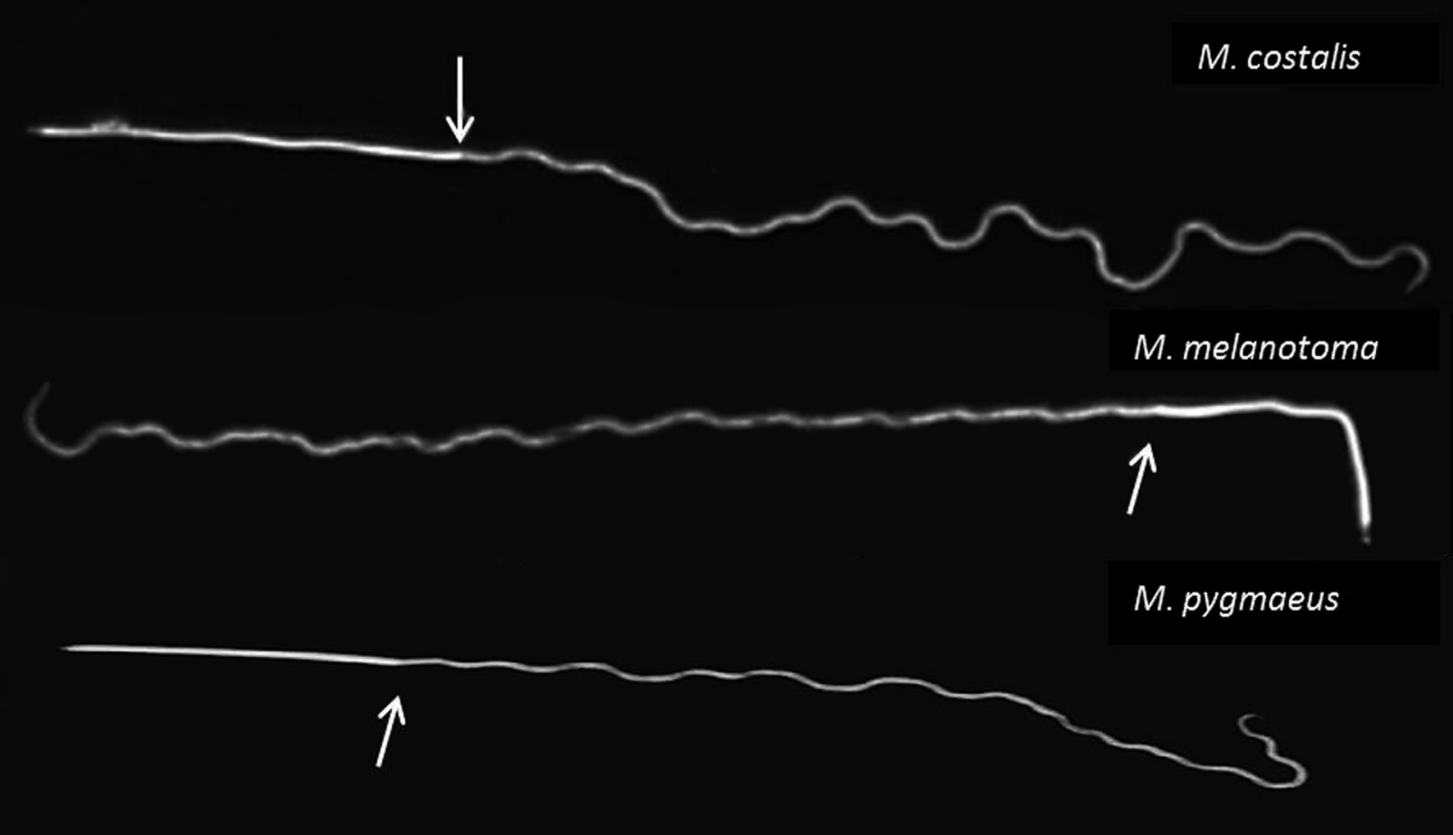
Sperm morphology of *Macrolophus* species. The arrow shows the end of sperm head.

Sperm cells of the species studied were of similar shape, with a long and filiform head (Fig. 18). However, species are different in the total length (mean ± SE) of the sperm cells (*F*_2,27_=15.53; *P*<0.0001), in the length of head (*F_2,27_*=38.89; *P*<0.0001) and tail (*F_2,27_*=25.43; *P*<0.01) (Table 1).

**Table 1.** Sperm cells lenght (means ± standard error) of *Macrolophus costalis*, *Macrolophus melanotoma* and *Macrolophus pygmaeus*. Within each row, means followed by the same letter do not differ significantly (P≤0.005).

Sperm length (µm)	*Macrolophus costalis*	*Macrolophus melanotoma*	*Macrolophus pygmaeus*
**Total**	236±0.9 **a**	220.4±0.9 **b**	215±1.4 **c**
**Head**	61.3±0.4 **a**	51.3±0.4 **b**	50.6±0.4 **b**
**Tail**	174.7±0.8 **a**	169.1±1 **b**	164.6±1.2 **c**

## Discussion

The genus *Macrolophus* belongs to the tribe Dicyphini of the subfamily Bryocorinae (Heteroptera, Miridae). In Bryocorinae, besides the modal for the Miridae chromosome number 2n=34 (32+XY), some higher (2n=36+XY and 2n=46+XY/X_1_X_2_Y) and lower (2n=16-26+XY) chromosome numbers have also been described (Ueshima 1979, Grozeva 2003, Grozeva et al. 2006, 2007, 2008a). The chromosome formula of the reference species *Macrolophus costalis* was reported earlier as 2n=28 (24+X_1_X_2_X_3_Y) for a Bulgarian population collected from the tobacco plants, which was the first report of three X chromosomes in the family (Grozeva et al. 2006). In contrast, the chromosome formula of the NE Spanish population here studied appeared to be different, with two instead of three X chromosomes. As the specimens come from different geographic regions we could speculate that they represent chromosomal races within a species. At taxonomic level, they may probably be considered as subspecies, but to clarify this hypothesis a chromosomal analysis of individuals (males and females) from natural populations of this species over the whole distribution range will be necessary. *Macrolophus pygmaeus* showed 2n=28 (26+XY) both in Bulgaria (Grozeva et al. 2007) and Spain (present study). The third species, *Macrolophus melanotoma*, studied here for the first time, appeared to differ from *Macrolophus costalis* and *Macrolophus pygmaeus* in karyotype. Besides difference in the chromosome number, *Macrolophus melanotoma* lacks two large autosome pairs characteristic of the two other species allowing for the cryptic *Macrolophus melanotoma* and *Macrolophus pygmaeus* to be reliably differentiated.

Differences in molecular organisation of chromatin revealed after fluorochrome staining, suggest an additional chromosome marker to differentiate *Macrolophus* species. In *Macrolophus costalis*, bright DAPI/CMA_3_ bands were observed in the same locations on the large autosomal bivalents and sex chromosomes whereas in two remaining species bright fluorescent bands were observed only on the sex chromosomes. In turn, *Macrolophus pygmaeus* differed from *Macrolophus melanotoma* in that it showed some additional weak DAPI-positive signals in a telomeric region of a larger bivalent.

Franco et al. (2011) have recently reported data on sperm morphology in *Macrolophus pygmaeus* and another dicyphine species *Nesidiocoris tenuis* Reuter, 1895. *Macrolophus pygmaeus* males were shown to have significantly smaller sperm cells (213.18 µm), with longer (50.94 µm) and wider heads and shorter tails (162.94 µm) than *Nesidiocoris tenuis*. In our study, the data on *Macrolophus pygmaeus* sperm cell size were confirmed. On the other hand, we found that *Macrolophus costalis* males have significantly longer sperm cells, with a longer head and tail compared to *Macrolophus melanotoma* and *Macrolophus pygmaeus*. In turn, *Macrolophus melanotoma* males have significantly longer sperm tails compared to *Macrolophus pygmaeus* (Table 1).

## Conclusion

As mentioned in Introduction, the cryptic species *Macrolophus pygmaeus* and *Macrolophus melanotoma* can be differentiated from each other based on the cuticular hydrocarbon profiles and specific molecular primers (Gemeno et al. 2012, Castañé et al. 2013). In our study, we provide some alternative characters, such as karyotype (number and size of chromosomes, sex chromosome system, and amount and distribution of heterochromatin) and sperm cells’ morphology, allowing for reliable identification of *Macrolophus pygmaeus*, *Macrolophus melanotoma* and *Macrolophus costalis*.
